# Metaheuristic Optimization-Based Feature Selection for Imagery and Arithmetic Tasks: An fNIRS Study

**DOI:** 10.3390/s23073714

**Published:** 2023-04-03

**Authors:** Amad Zafar, Shaik Javeed Hussain, Muhammad Umair Ali, Seung Won Lee

**Affiliations:** 1 Department of Intelligent Mechatronics Engineering, Sejong University, Seoul 05006, Republic of Korea; 2 Department of Electrical and Electronics, Global College of Engineering and Technology, Muscat 112, Oman; 3 Department of Precision Medicine, School of Medicine, Sungkyunkwan University, Suwon 16419, Republic of Korea

**Keywords:** motor imagery, mental arithmetic, fNIRS, feature selection, optimization, brain–computer interface (BCI)

## Abstract

In recent decades, the brain–computer interface (BCI) has emerged as a leading area of research. The feature selection is vital to reduce the dataset’s dimensionality, increase the computing effectiveness, and enhance the BCI’s performance. Using activity-related features leads to a high classification rate among the desired tasks. This study presents a wrapper-based metaheuristic feature selection framework for BCI applications using functional near-infrared spectroscopy (fNIRS). Here, the temporal statistical features (i.e., the mean, slope, maximum, skewness, and kurtosis) were computed from all the available channels to form a training vector. Seven metaheuristic optimization algorithms were tested for their classification performance using a k-nearest neighbor-based cost function: particle swarm optimization, cuckoo search optimization, the firefly algorithm, the bat algorithm, flower pollination optimization, whale optimization, and grey wolf optimization (GWO). The presented approach was validated based on an available online dataset of motor imagery (MI) and mental arithmetic (MA) tasks from 29 healthy subjects. The results showed that the classification accuracy was significantly improved by utilizing the features selected from the metaheuristic optimization algorithms relative to those obtained from the full set of features. All of the abovementioned metaheuristic algorithms improved the classification accuracy and reduced the feature vector size. The GWO yielded the highest average classification rates (*p* < 0.01) of 94.83 ± 5.5%, 92.57 ± 6.9%, and 85.66 ± 7.3% for the MA, MI, and four-class (left- and right-hand MI, MA, and baseline) tasks, respectively. The presented framework may be helpful in the training phase for selecting the appropriate features for robust fNIRS-based BCI applications.

## 1. Introduction

Brain–computer interface (BCI) technology enables a direct connection between the brain and an external device by avoiding the use of traditional channels, such as peripheral nerves and muscles [1]. BCIs are designed to provide impaired people (such as those with locked-in syndrome or spinal cord injuries) with new ways to communicate and manage their surroundings. BCIs may also be employed in industries such as gaming, transportation [2], recreation [3], virtual reality, human–machine interfaces [4], and neurological rehabilitation [5,6,7,8]. BCIs are classified into two types: invasive and non-invasive. Invasive BCIs capture brain activity using electrodes placed directly into the brain. This approach provides the highest-level information for signals but possesses significant risks, including the chance of infection and lasting brain tissue damage. Non-invasive BCIs, by contrast, do not require electrodes to be implanted in the brain. Instead, they capture signals from the scalp or other areas of the head using methods, such as electroencephalography (EEG) [9,10], functional magnetic resonance imaging [11,12], magnetoencephalography [13], and functional near-infrared spectroscopy (fNIRS) [14,15]. Non-invasive BCIs are less dangerous and less obtrusive than invasive BCIs but provide lower-resolution signals. The choice between invasive and non-invasive BCIs is determined by the specific application and the trade-off between the resolution and invasiveness. Despite these potential benefits, significant technical and clinical hurdles must be overcome before BCIs can be widely adopted and utilized.

Each non-invasive BCI has its own advantages and disadvantages. As a relatively new technique, fNIRS offers a balance between the temporal and spatial resolution and a variety of distinctive benefits [16,17,18]. Oxyhemoglobin (HbO/∆HbO) and deoxyhemoglobin (HbR/∆HbR) concentration changes are measured using fNIRS, utilizing pairs of multiple near-infrared lights (650–1000 nm range) that penetrate through the superficial cortical areas. Both the absolute and relative concentration changes are measured [19,20]. The neocortex activation caused by the brain stimulation results in increased blood flow and oxygenation levels (as reflected by the increases in ∆HbO or the decreases in ∆HbR). These modifications can then be used to provide control signals for the fNIRS-based BCI applications.

The development of rapid systems for quicker command decoding is one of the three primary research objectives in the field of BCIs. The other two are maximizing the number of decoded commands and improving the classification performance. The features are extracted using various small window sizes (0–2, 0–2.5, 0–5, 2–7, etc.) to speed up the BCI system [21,22,23]. fNIRS is used with other measuring modalities (e.g., EEG) to increase the number of commands that can be decoded from the brain [24,25]. The selection of the channels and features is a key component for enhancing the BCI classification accuracy. In fNIRS-based BCI research, active channel selection has been performed using various methods, such as averaging on a particular region of interest [26,27], computing the Pearson correlation coefficient [28], performing vector phase analyses [21,29,30,31], averaging across all the channels [32,33,34], applying baseline corrections [24], calculating the contrast-to-noise ratios [35], using t-statistics and z-statistics [23,30,36], using a least absolute shrinkage and selection operator homotopy-based sparse representation [37], and utilizing joint-channel-connectivity methods [38].

Various types of features have been used to decode fNIRS signals in the literature, including statistical features [39], graph-based features [40], Mel frequency cepstral coefficients [41], vector phase analysis-based features [29], and frequency domain-based features [42]. Different ranges of two- and three-feature combinations have been used in prior studies by using temporal statistical data to determine the best combinations for classifying the various activities [43,44]. However, selecting the best features using visual inspection can be challenging, particularly when all the channels are used for the feature extraction and classification. Various studies have proven the impact of the feature selection on BCIs [29,45,46]. It helps to reduce the dataset’s dimensionality, increase the computing effectiveness, and enhance the classification performance among the tasks. By identifying the features that are related to the activity, the feature selection increases the robustness of the classification system. Therefore, in one study [47], the authors used a genetic algorithm (GA) to determine the best window size for the computation of the temporal features. Their suggested framework improved the model’s capability for classification. A recent study [45] presented a systematic approach for choosing the subject-specific features. They used filter-based techniques to remove the redundant features and boost the classification efficiency. Similarly, the “ReliefF” filter merged with a GA was used to classify upper limb movement intentions [48]. In another study, a minimum-redundancy maximum-relevance filter was used to reduce the feature vector size and enhance the classification performance. The relevant features were also identified using a sparse representation classification method. [49]. Recently, Dokeroglu et al. [50] reviewed various wrapper-based feature selection methods. Wrapper approaches examine the performance of each subset of features and combine a metaheuristic optimization algorithm with a classifier. Except for GAs, other wrapper-based feature selection methods have not been explored for fNIRS-based BCIs in the literature. The no-free-lunch (NFL) theorem states that no heuristic is sufficient to address every optimization issue. Most metaheuristics excel in at least one area compared to the others. The optimal subset of the features from several domains may not always be discovered using a single metaheuristic algorithm.

Therefore, herein, wrapper-based approaches operating in conjunction with metaheuristic optimization algorithms were explored for an fNIRS BCI to improve the accuracy and accelerate the processing. The following are the main highlights of the presented work.

- First, the data of all the channels of the fNIRS signals were pre-processed to remove physiological noise.
- The most commonly used statistical temporal features were computed for the fNIRS signals in all the channels.
- Wrapper approaches with various metaheuristic optimization algorithms, such as particle swarm optimization (PSO), cuckoo search optimization (CSO), the firefly algorithm (FA), the bat algorithm (BA), flower pollination optimization (FPO), the whale optimization algorithm (WOA), and grey wolf optimization (GWO), were applied to observe the classification performance using a k-nearest neighbor (k-NN) approach.
- The performance of the proposed framework was evaluated using the online motor imagery (MI) and mental arithmetic (MA) datasets.
- A statistical analysis was also performed to determine the significance of the obtained results.

## 2. Proposed Framework

**Figure 1:**
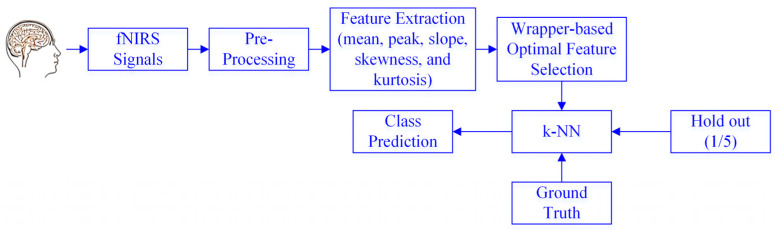
Proposed wrapper-based feature selection approach for the functional near-infrared spectroscopy (fNIRS) signals.

This section explains a framework for the feature selection for the fNIRS signals using optimization algorithms. This framework entailed choosing the most pertinent and significant features from the fNIRS signals. Here, the acquired fNIRS data were pre-processed to remove the physiological noise, and further details are provided in Section 3. After that, the temporal statistical features were extracted for all the channels in a 10-s window. A wrapper-based feature selection method was applied to retrieve the most important features using PSO, CSO, the FA, the BA, FPO, the WOA, and GWO. All the algorithms were implemented using “Jx-WFST,” a Jxwrapper feature selection toolbox in MATLAB (https://www.mathworks.com/matlabcentral/fileexchange/84139-wrapper-feature-selection-toolbox, accessed on 7 February 2023). The k-NN model was selected for the classification, as it is a non-parametric machine learning approach that is accurate, easy, and widely used [51,52]. In this approach, the neighbors’ votes determine how a sample is classified. The item corresponding to the *k* training samples (i.e., *k*, the object’s closest neighbors) is categorized into a class based on the greatest class probability [53]. Further details on the k-NN can be found in [54]. The framework of the proposed approach is shown in Figure 1.

## 3. Experimental Data and Pre-Processing

**Figure 2:**
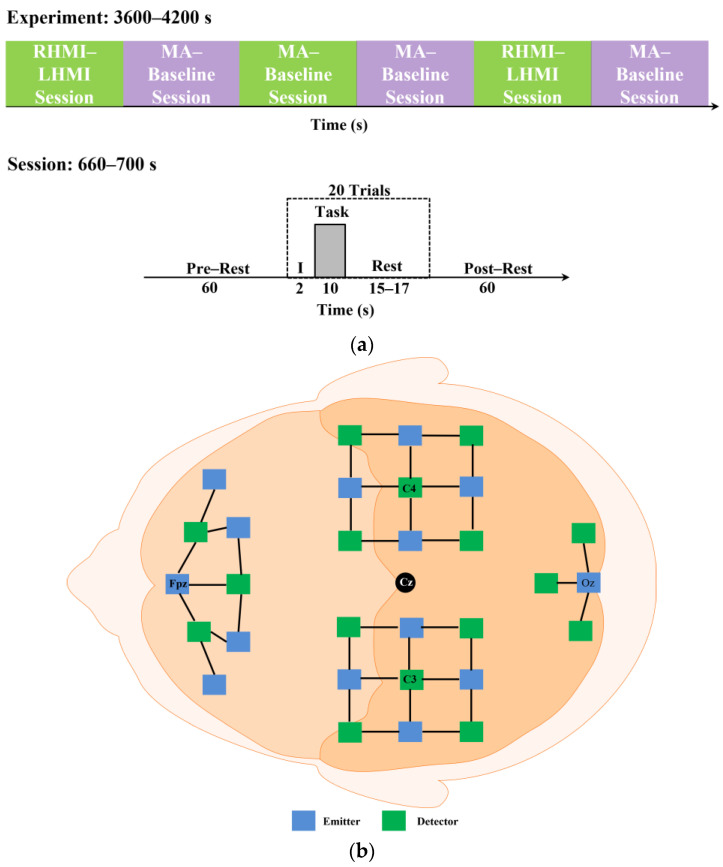
(**a**) Experimental paradigm; (**b**) fNIRS optodes placement.

Here, the fNIRS data of 29 participants from an online dataset were used to validate the presented methodology [55]. The prefrontal, motor, and occipital brain regions were surrounded by 36 channels formed by fourteen sources and sixteen detectors spaced three centimeters apart, utilizing the 10-5 international system with Fp1, Fp2, Fpz, C3, C4, and Oz as the references. The fNIRS data were measured at a 12.5 Hz sampling rate and were down-sampled to 10 Hz. The dataset was composed of triggered, fNIRS, and EEG data from six different sessions. The dataset was divided into datasets A and B corresponding to the left-hand motor imagery (LHMI) and right-hand motor imagery (RHMI) sessions (i.e., 1, 3, and 5) as well as the MA and baseline sessions (i.e., 2, 4, and 6). There was a 1-min pre-rest time at the start of each session, followed by 20 trials (10 for each activity) and followed by another 1-min post-rest interval. The exercise consisted of a 2-s visual introduction, a 10-s task phase, and a rest period randomly allocated to last between 15 and 17 s. Here, only the fNIRS and associated trigger data were used. The fNIRS data (i.e., only ΔHbO in this study) were passed through a 3rd-order Butterworth low-pass filter with a 0.1 Hz cutoff frequency and a Butterworth high-pass filter with a 0.01 Hz cutoff frequency to reduce the physiological noise. Figure 2 shows the fNIRS optode placement and the experimental paradigm [55].

## 4. Feature Extraction

The fNIRS trials for both the MI and MA were retrieved after pre-processing, and a section of the trial lasting 10 s was utilized for the feature extraction. In this investigation, only the top five most commonly used statistical features—the mean, peak, slope, skewness, and kurtosis—were retrieved. [39,56,57,58]. The mean, skewness, and kurtosis were determined using the following formulas, wherein the peak value is the maximum value, and the slope is retrieved using the curve fitting.

(1) $$\mu =\frac{1}{N}\sum_{k=k_{1}}^{k_{2}}X(k)$$

(2) $$S_{x}=\frac{E_{x}{\left(X_{x}-\mu_{x}\right)}^{3}}{\sigma^{3}}$$

(3) $$K_{x}=\frac{E_{x}{\left(X_{x}-\mu_{x}\right)}^{4}}{\sigma^{4}}$$

In the above, X represents the signals of the fNIRS (i.e., ΔHbO) and μ and σ denote the mean and standard deviation, respectively. $S_{x}$, $K_{x}$, and $E_{x}$ are the skewness, kurtosis, and expectation of X, respectively.

Herein, initially, all of the fNIRS channels were used for the feature extraction. In total, 180 features (36 × 5) were retrieved for a single trial. Thus, the feature selection was necessary to choose the right features, further minimize the feature vector size, and enhance the classification performance.

## 5. Feature Selection Method

In the feature selection process, a subset was chosen from the larger set of features to develop the machine learning model. The quality of the created candidate subsets was assessed using a predetermined criterion [59]. The feature selection aimed to improve the model’s performance, decrease overfitting, and enhance the interpretability. The test classification accuracy was used to validate the outcome of the feature selection method. In general, feature selection algorithms may be divided into three primary categories, i.e., filter, embedding, and wrapper approaches, depending on the numerous assessment criteria and techniques used to generate the subsets.

Following the theory that the features with a high variance provide the most relevant details, the filter feature techniques are geared to maintain the features with a more significant variance [60]. These methods typically take little time to execute, although they can select redundant variables.

Embedding methods do not distinguish between the feature selection procedure and the classification method [61]. The feature selection is conducted within the classification process; therefore, it is incorporated as an algorithmic component or a functionality enhancement.

**Figure 3:**
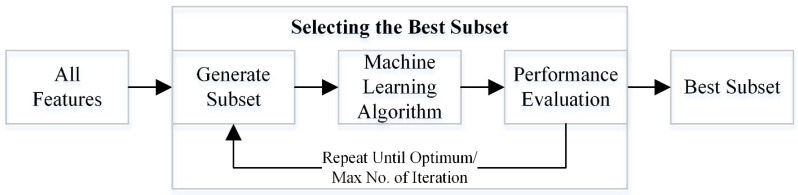
Schematic of the working of the wrapper-based method.

Wrapper algorithms are machine learning techniques that assess the performance of a subset of features using a particular machine learning model (the “wrapper”) [62]. The wrapper’s objective is to assess how the chosen features affect the model’s performance. Based on the evaluation findings, the wrapper algorithm will either choose the current subset of features or search for a better subset of features. The best subset of features is eventually discovered by repeating this approach. The wrapper approach concept is presented in Figure 3. This work uses wrapper-based techniques and various metaheuristics algorithms for the optimal feature selection.

### 5.1. Metaheuristics Algorithms for Wrapper-Based Methods

Metaheuristic algorithms aim to approximate solutions to challenging problems. They are called “meta” because they manage high-level optimization problems by integrating several low-level heuristics [63,64]. PSO, CSO, the FA, the BA, FPO, the WOA, and GWO, among others, are examples of metaheuristic algorithms [50,65].

Learning algorithms are used in wrapper feature selection approaches to assess the classification performance of the generated feature subsets. The metaheuristics serve as the search algorithms to generate new potential optimal subsets [66]. The cost function for all of the optimization algorithms is defined as shown in Equation (4) [67].

(4) $$\min(J)=\alpha (1-Accuracy)+\beta \left(\frac{no.\;of\;selected\;features}{no.\;of\;total\;features}\right)$$

Here, α and β are selected with the default values of 0.99 and 0.01, respectively.

#### 5.1.1. Particle Swarm Optimization (PSO)

PSO is a technique for solving complicated optimization issues. It depends on the coordinated behavior of a collection of particles traveling through a solution space while being directed by the experiences of their nearby neighbors to locate the best solution [68,69]. It is inspired by the coordinated activity of a flock of birds or school of fish. Each particle in PSO represents a potential solution to the issue, is initially placed, and moves randomly. The particles adjust their locations (X) after each iteration depending on their current velocity (V) and considering their own personal best solution (pbest) and the group’s overall best solution (gbest). The pull toward the pbest and gbest solutions and a random element to promote exploration are combined with the particle’s current velocity to create a velocity update. The process is continued until a stopping requirement is satisfied, e.g., achieving a suitable solution quality or completing a predetermined number of iterations [70]. The equations below can be used to determine each particle’s velocity and update its position.

(5) $$\begin{array}{l} V_{ij}^{t}=\alpha V_{ij}^{t-1}+a_{1}b_{1j}^{t-1}\left[\mathrm{pbest}-X_{ij}^{t-1}\right]+a_{2}b_{2j}^{t-1}\left[\mathrm{gbest}-X_{ij}^{t-1}\right]\\ X_{ij}^{t}=X_{ij}^{t-1}+V_{ij}^{t} \end{array}\}$$

where α, $a_{1}$, $a_{2}$, $b_{1}$, and $b_{2}$ are the parameters of PSO. The $b_{1}$ and $b_{2}$ can be randomly selected. Further details about the PSO can be found in [71]. Algorithm 1 shows the pseudo code of PSO.
**Algorithm 1** Pseudo code of PSO [71].1. Generate a random population of particles (*N*)

2. **while** the termination condition is not satisfied **do**

3. **for** each particle **do**
4. Evaluate the fitness of all the particles using the fitness function

5. Update the velocity and position of all the particles using Equation (5)

6. Evaluate the fitness $f({\mathrm{Pr}}_{i}^{j})$

7. **$\mathrm{if}\;f({\mathrm{Pr}}_{i}^{t})<f(pbest_{i}^{t})$ then**

8. $pbest_{i}^{t}\leftarrow {\mathrm{Pr}}_{i}^{t}$

9. **if** $f({\mathrm{Pr}}_{i}^{t})<f(gbest_{i}^{t})$ **then**

10. $gbest_{i}^{t}\leftarrow {\mathrm{Pr}}_{i}^{t}$

11. **return** gbest


#### 5.1.2. Cuckoo Search Optimization (CSO)

The CSO metaheuristic optimization method was developed in response to the cuckoo bird’s tendency to lay its eggs in the nests of other bird species. The CSO algorithm was proposed by Yang in 2009 [72]. In CSO, a population of potential solutions to a problem is retained and an algorithm that uses random walk and exploitation operations iteratively improves it. The cuckoo’s propensity to deposit its eggs in other birds’ nests served as the inspiration for the random walk operation, which represents the discovery of new solution areas. The algorithm can concentrate on and enhance the most promising solutions with the help of the exploitation operation. In contrast to the other optimization algorithms, CSO finds high-quality solutions more quickly owing to its careful balance between exploration and exploitation. Additionally, CSO may be quickly parallelized for complicated optimization issues and is very easy to implement. It also does not require extensive parameter adjustment. The following equations can be utilized to model CSO.

(6) $$\begin{array}{l} x_{i}^{t+1}=x_{i}^{t}+\alpha Levy(\lambda )\\ Levy∼u=t^{-\lambda } \end{array}\}$$

where $x_{i}$ is the solution at the step size (α) and λ is the variance of the levy distribution. Further details about CSO can be found in [73]. Algorithm 2 shows the pseudo code of CSO.
**Algorithm 2** Pseudo code of CSO [73]
1. Generate a random population of host nests (*N*)
2. **while** the termination condition is not satisfied **do**

3. Select a random cuckoo (*i*) and determine a new solution using Equation (6)

4. Evaluate the fitness using the fitness function; *F_i_*

5. Randomly choose a nest (*j*) from the population

6. $\mathrm{if}\;(F_{i}>F_{j})$ **then**

7. Replace *j* with a new solution
8. **end if**
9. Abandon a fraction (*P*_a_) of the worst nest and a new one at new location using Levy flights

10. Rank the solution and find the current best

11. **return** $S_{best}$


#### 5.1.3. Firefly Algorithm (FA)

In 2010, Yang presented the FA, which was motivated by the flashing qualities of fireflies [74]. These flashes draw potential mates or warn off predators. In the FA, the flashing properties are developed and applied as functions to solve combinatorial optimization issues [75]. According to their levels of brightness, fireflies attract other flies and advance toward brighter fireflies. The more space between the fireflies, the less appealing they become. The fireflies move at random when a brighter one is not present. As a result, the critical influences on the FA are the light intensity and the attractiveness level. A selected function can be used to control the brightness at a particular place. As it relies on the distance and absorption coefficient, the attraction is determined by the other fireflies. The FA can be modeled as follows.

(7) $$\begin{array}{l} I=I_{o}e^{-\gamma r}\\ \beta =\beta_{o}e^{-\gamma r^{2}}\\ x_{i}^{t}=x_{i}^{t-1}+\beta_{o}e^{-\gamma r_{ij}^{2}}(x_{j}^{t-1}-x_{i}^{t-1})+\alpha^{t}\in_{i}^{t} \end{array}\}$$

where I and $I_{o}$ are the current and default light intensities; γ and r are the light absorption coefficient and the distance, respectively; β and $\beta_{o}$ are the current and default attractiveness (when the distance is zero); $x_{i}$ is the position; j is the higher intensity firefly; and α and ∈ are the random parameters. Further details about the FA can be found in [76]. Algorithm 3 shows the pseudo code of the FA.
**Algorithm 3** Pseudo code of the FA [76]
1. Generate a random population of fireflies (*N*)

2. Evaluated the fitness value *f*(*x_i_*)
3. Initialize the parameters *T*, γ

4. **while** (*t* < *T*) **do**

5. **for** *i* = 1 to *N* **do**

6. **for** *j* = 1 to *i* **do**

7. $\mathrm{if}\;(I_{j}<I_{i})$ **then**

8. Compute the attractiveness and move firefly *i* towards *j* using Equation (7)

9. **end if**
10. **end for**

11. **end for**

12. Evaluate the fitness value

13. Rank the fireflies and determine the current best

14. **end while**

#### 5.1.4. Bat Algorithm (BA)

The BA is a nature-inspired optimization algorithm introduced by Yang in 2010 [77]. It is based on how bats use echolocation to locate their prey. The method is modeled after how bats fly around randomly while looking for food, generating noises, and listening for echoes to find their meal [78]. Using random walk and exploitation procedures, the BA maintains a population of alternative solutions to an optimization issue and iteratively enhances these solutions. While the exploitation operation enables the algorithm to concentrate on the most promising answers, the random walk operation imitates the unpredictable search behaviors of bats. Its usage of a frequency-tuning mechanism is motivated by the frequency modulation of bat sounds. This mechanism allows the algorithm to break out of the local optima and discover superior solutions. Furthermore, the BA is easy to deploy and does not require complicated parameter tuning. It is possible to codify echolocation as a method for improving an objective function [79]. The equations below can be used to model this algorithm.

(8) $$\begin{array}{l} f_{i}=f_{\min}+(f_{\max}-f_{\min})\beta \\ v_{i}^{t}=v_{i}^{t-1}+(x_{i}^{t}-x_{*})f_{i}\\ x_{i}^{t}=x_{i}^{t-1}+v_{i}^{t}\\ x_{new}=x_{old}+\alpha A^{t} \end{array}\}$$

where $x_{i}$ and $v_{i}$ are the position and velocity in the frequency range between $f_{\max}$ and $f_{\min}$; β is the random vector; $x_{*}$ is the best available solution; and α and $A^{t}$ are the random vector between the range of [0, 1] and the maximum loudness of the bats. Further details about the BA can be found in [80]. Algorithm 4 shows the pseudo code of the BA.
**Algorithm 4** Pseudo code of the BA [80]
1. Generate a random population of bats (*N*) and an initial velocity *v*_i_

2. Evaluated the fitness value *f*(*x_i_*)

3. Define *f_i_*
4. Initialize the parameters *T*, α, and $A^{t}$

5. **while** (*t* < *T*) **do**

6. Update the frequency, velocity, and position using Equation (8)

7. **$\mathrm{if}\;(rand<r_{i})$ then**

8. Select the best result and create a local result around the best result

9. **end if**

10. $\mathrm{if}\;(rand<A_{i})\;and\;(f(x_{i})<f(x^{*}))$ **then**

11. Store the new result, decrease *A*_i_, and increase *r*_i_

12. **end if**

13. Rank the bats and determine the current best

14. **end while**

#### 5.1.5. Flower Pollination Optimization (FPO)

The metaheuristic optimization method known as FPO was motivated by the pollination process in flowers [81]. It was developed to address the optimization issues in various fields, including computer science, engineering, mathematics, physics, and finance. The optimization procedure in FPO is based on how pollen moves between flowers. The quality of a solution is expressed by how much “nectar” it contains, and each solution in the optimization problem is represented as a “flower.” The nectar is transferred between the flowers in a way that is similar to how the optimization problem solutions are selected and merged. FPO is renowned for its ease of use, quick convergence speed, and simplicity. It has been used to address numerous optimization issues, including function optimization, scheduling, and data clustering issues [82]. FPO can be modeled using the following equations.

(9) $$\begin{array}{l} x_{i}^{t}=x_{i}^{t-1}+\alpha L(\lambda )(g_{*}-x_{i}^{t-1})\\ L(\lambda )=\frac{\lambda \Gamma (\lambda )\cdot \sin(\lambda )}{\pi }\cdot \frac{1}{s^{1+\lambda }}\\ x_{i}^{t}=x_{i}^{t-1}+\varepsilon (x_{j}^{t-1}-x_{k}^{t-1}) \end{array}\}$$

where $x_{i}^{t}$ is the pollen; $g_{*}$ is the best available solution; α is the scaling factor [0, 1]; L(λ) and Γ(λ) are the levy step size and gamma function; and $x_{j}^{}$ and $x_{k}^{}$ are the pollen from the different flowers but the same plant species. Further details about FPO can be found in [83]. Algorithm 5 shows the pseudo code of FPO.
**Algorithm 5** Pseudo code of FPO [83]
1. Generate random pollinators and flowers

2. Initialize the parameters

3. Evaluate the solution of the population using the fitness function *f*(*x*)

4. Determine the global best solution (g_*_)

5. **while** (*t* < *T*) **do**

6. **for** *i* = 1:N

7. **if** (*p* < rand) **then**

8. Draw a (d-dimensional) step vector L that obeys a Levy distribution and perform global pollination

9. **else**

10. Pick *ε* randomly [0–1]
11. Randomly choose *j* and *k* among all the solutions and perform local pollination

12. **end if**

13. Evaluate *f*_min_ of the new solution

14. If *f*_min_ is better than previous solution, update the *i* solution and *f*_min_ in the population, and if *f*_min_ is better than global best solution, update the global best solution and its *f*_min_

15. **end for**

16. Store the global best solution

17. *t* = *t* + 1

18. **end while**

#### 5.1.6. Whale Optimization Algorithm (WOA)

In 2016, Mirjalili and Lewis [84] introduced the WOA, which is inspired by humpback whales’ hunting habits. These whales hunt in groups, demonstrating their gregarious nature. They blow bubble nets to catch their prey (such as tiny fish or krill) when they come across clusters of prey. The WOA mathematical model represents a fresh approach to addressing complex optimization issues. The algorithm’s primary tasks are finding prey, surrounding it, and moving in spiral bubble-net patterns. The WOA uses a variety of strategies after starting with a random solution. The other agents adjust their places after choosing the top search agent. They choose a target (either the best search agent or a random whale) and proceed to attack it. The WOA has helped resolve various optimization issues, including function optimization, scheduling, and data clustering issues [50]. The following equations can be used to model the WOA.

(10) $$\begin{array}{l} {\vec{x}}_{i}^{t}=\vec{D}{}^{\prime }\cdot e^{bl}\cdot \cos(2\pi l)+x^{*}{}_{i}^{t-1}\\ \vec{D}{}^{\prime }=\left|x^{*}{}_{i}^{t-1}-{\vec{x}}_{i}^{t-1}\right| \end{array}\}$$

where $\vec{x}$ and $x^{*}$ are the current and best locations, respectively; b and l are the constant for determining the logarithmic spiral shape and a random number, respectively; and $\vec{D}{}^{\prime }$ is the distance between the whale and the prey. Further details about the WOA can be found in [85,86]. Algorithm 6 shows the pseudo code of the WOA.
**Algorithm 6** Pseudo code of the WOA [86]
1. Generate a random population (*N*)

2. Initialize the parameters

3. Evaluate the solution of the population using the fitness function

4. Determine the best solution (x*)

5. **while** (*t* < *T*) **do**

6. **for** each solution **do**

7. Update all the parameters (i.e., *a*, *A*, *C*, l, and *p*)

8. **if** (*p* < 0.5) **then**

9. **if** |*A*| < 1 **then**

10. Update the position of the current solution by using $\vec{x}(t+1)={\vec{x}}^{*}(t)-\vec{A}\cdot D$

11. **else if** |*A*| > 1 **then**

12. Select a random solution from a population

13. Update the position of x(t) by using $\vec{x}(t+1)={\vec{x}}_{rand}-\vec{A}\cdot \vec{D}$

14. **end if**

15. **else if** (*p* > 0.5) **then**

16.Update the position of *x*(*t*) using Equation (10)

17. **end if**

18. **end for**

19. Evaluate the fitness value for each solution.

20. Update x*

21. *t* = *t* + 1

22. **end while**

#### 5.1.7. Grey Wolf Optimization (GWO)

The GWO was presented by Mirjalili et al. in 2014 [87] and its multi-objective variant was introduced in 2016 [88]. Predators such grey wolves live and hunt in groups. Grey wolves live in social packs composed of five to twelve individuals. Depending on their level of authority over others, the group members are referred to as alpha, beta, omega, or subordinates in this social system. Each pack has a single alpha wolf or a dominant wolf and leader of the group. As a result, the alpha is in charge of the majority of tasks. The second most dominating wolf is the beta. It is anticipated that the beta will become the dominant wolf (alpha). The beta supports the alpha’s decision-making, communicates his/her orders to the group, and sees them through. The lowest ranking wolf in the group (ruled by all the other wolves) is named the omega. The remaining wolves are referred to as subordinate or delta wolves. In addition to this social organization, grey wolves also hunt in groups. The group tracks and pursues a victim when it is within range. They surround the target whenever feasible, then each attacks in turn. The grey wolves’ characteristics are considered for modeling GWO. The mathematical representation of GWO comprises the social structure and prey hunting techniques. The alpha, beta, and delta wolves in GWO are chosen as the top three solutions. The other wolves in the pack are considered the omega wolves and do not influence the choices made for the following iteration. The GWO technique was created to address the optimization issues prevalent in many disciplines, including computer science, engineering, mathematics, physics, and finance [67,89]. The variants of GWO are also proposed and applied to solve various engineering problems [90,91]. GWO can be modeled using the following equations.

(11) $$\begin{array}{l} {\vec{x}}^{t}={\vec{x}}_{p}^{t-1}+\vec{A}\cdot \vec{D}\\ \vec{D}=\left|\vec{C}\cdot {\vec{x}}_{p}^{t-1}-{\vec{x}}^{t}\right|\\ \vec{A}=2a\cdot {\vec{r}}_{1}-a\\ \vec{C}=2{\vec{r}}_{2} \end{array}\}$$

where $\vec{x}$ and ${\vec{x}}_{p}$ are the grey wolf and prey locations, respectively; $\vec{A}$ and $\vec{C}$ are the coefficient vectors; $\vec{D}{}^{\prime }$ is the distance between the grey wolf and the prey; $r_{1}$ and $r_{2}$ are the random vectors; and a is the linearly decreased variable. It can be calculated using the following equation.

(12) $$a=2-t\frac{2}{\max\mathrm{iterations}}$$

where is the iteration number. The following equations can be used to update the grey wolves’ position.

(13) $${\vec{x}}^{t}=\frac{{\vec{x}}_{1}+{\vec{x}}_{2}+{\vec{x}}_{3}}{3}$$

where

(14) $$\begin{array}{l} {\vec{x}}_{1}=\left|{\vec{x}}_{\alpha }-{\vec{A}}_{1}{\vec{D}}_{\alpha }\right|\\ {\vec{x}}_{2}=\left|{\vec{x}}_{\beta }-{\vec{A}}_{2}{\vec{D}}_{\beta }\right|\\ {\vec{x}}_{3}=\left|{\vec{x}}_{\delta }-{\vec{A}}_{3}{\vec{D}}_{\delta }\right|\\ {\vec{D}}_{\alpha }=\left|{\vec{C}}_{1}\cdot {\vec{x}}_{\alpha }-\vec{x}\right|\\ {\vec{D}}_{\beta }=\left|{\vec{C}}_{2}\cdot {\vec{x}}_{\beta }-\vec{x}\right|\\ {\vec{D}}_{\delta }=\left|{\vec{C}}_{3}\cdot {\vec{x}}_{\delta }-\vec{x}\right| \end{array}\}$$

Further details about the GWO can be found in [92]. Algorithm 7 shows the pseudo code of GWO.
**Algorithm 7** Pseudo code of GWO [92]
1. Generate a random population (*N*)

2. Initialize the parameters

3. Evaluate the fitness of each wolf

4. **while** (*t* < *T*) **do**

5. **for** each wolf **do**

6. Update the position using Equations (13) and (14)

7. **end for**

8. Update the parameters

9. Evaluate the fitness value

10. Update the wolf’s position

11. *t* = *t* + 1

12. **end while**

13. **return**
${\vec{x}}_{\alpha }$

## 6. Results and Discussion

This study applied wrapper-based metaheuristics feature selection approaches to enhance the fNIRS signals’ classification accuracy. The MA and MI datasets were used to test the suggested feature selection algorithms [55]. The discriminations between the MA and baseline, LHMI and RHMI, and LHMI, RHMI, MA, and baseline were accomplished by utilizing a feature set composed of five statistical temporal features, as discussed above. The wrapper-based metaheuristic algorithms discussed above (PSO, CSO, the FA, the BA, FPO, the WOA, and GWO) were employed to extract valuable information. The extracted feature subset was classified using a k-NN and a 0.2-holdout validation technique. The *K* parameter of the k-NN was set as 5, N was the population size and *T* was the maximum number of iterations. Each algorithm was run ten times. All the parameters for each algorithm are enlisted in Table 1.

The results from all three cases are presented in Table 2, Table 3 and Table 4. This study used the classification accuracy and the feature vector size as the performance comparison metrics.

Table 2, Table 3 and Table 4 present the results for all three cases with each optimization algorithm for the specific subjects, using the accuracy and the feature vector size as the comparison metrics. In the case of the MA tasks, the full feature vector (180 features for each task) resulted in a 91.67% classification accuracy for subject one. By contrast, all of the wrapper-based optimization algorithms had a higher classification accuracy with a significantly reduced feature vector size, as shown in Table 2. After carefully analyzing the results, it can be seen that the GWO algorithm had a 99.1 ± 2.6 classification rate with a feature vector size of only 24.1 ± 5.1 for subject one. The algorithm enhanced the classification rate more than 8% from almost 150 fewer features. The WOA algorithm used the lowest number of features for the classification and produced a good classification rate when using all the channel features. For instance, in the cases of subjects three and four, the WOA only utilized 12.5 ± 14 and 4.8 ± 2.8 features to classify the MA task with the baseline and produced classification rates of 81.6 ± 8.6% and 88.3 ± 8.9%, respectively. These were almost similar results to those from the cases using all the channel features.

In the case of the MI tasks, the full channel features yielded an accuracy of only 41.67% for subject one. By contrast, the WOA produced the highest classification rate of 92.5 ± 8.2 with 35.7 ± 13.7 features. The FA showed an 85.8 ± 8.8 classification performance for subject 14 with 80.5 ± 6.8 features. The BA showed an 84.1 ± 8.2 classification rate for subject six with 75.2 ± 6.3 features, i.e., 10% more than using all the features. The results for the other subjects for the MI classification tasks are shown in Table 3.

**Figure 4:**
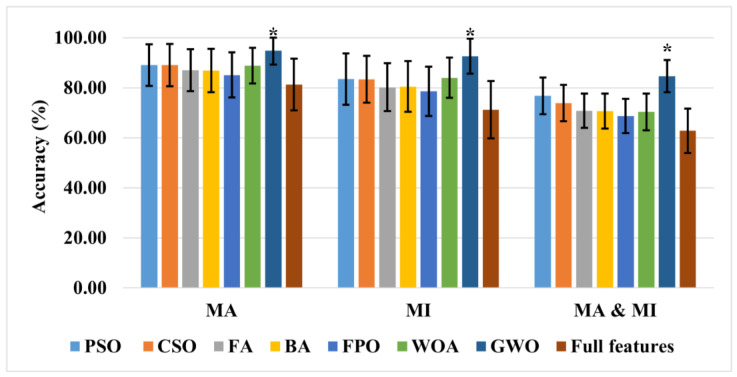
Comparison of all the presented metaheuristic algorithms and all the channel features for the various tasks (data represented as the mean ± the standard deviation). * *p* < 0.01.

Table 4 shows the results from the merged MA and MI datasets. This was performed to observe the proposed strategy’s effectiveness in a multi-class environment. As depicted in Table 4, the accuracy when using all the features was reduced for all of the subjects compared to the MA task alone. The results presented in Table 4 demonstrate the effectiveness of the proposed approach in a multi-class environment. For a better understanding of the results, the average classification accuracies, processing times, and feature vector sizes of all the subjects for the various tasks are presented in Figure 4 and Table 5.

After deeply analyzing the results, it can be seen that GWO produced the highest classification rates of 94.83 ± 5.5%, 92.57 ± 6.9%, and 85.66 ± 7.3% for the MA, MI, and four-class (left- and right-hand MI, MA, and baseline) tasks, respectively. All of the channel features had classification rates of only 81.32%, 71.26%, and 62.79% for similar tasks. All of the optimization algorithms significantly improved the classification rate and reduced the feature vector size. However, GWO produced the best and most stable results since it was scalable, versatile, simple to use, and didn’t need any derivation information from the search space. The search method for the algorithm benefitted from a balance between exploration and exploitation, producing an excellent convergence [93].

A two-sample *t*-test was also utilized to determine the statistical significance of the results. The results of GWO remained true compared to those from the full feature set and all the other optimization methods’ results (*p* < 0.01). Table 5 presents the average processing time of each algorithm. The WOA had the lowest processing time and the smallest feature vector size with a reasonable accuracy. GWO used 29 features on average with only 2.17 s of processing time to yield the highest classification accuracy, as shown in Figure 4 and Table 5. As shown in Table 6, the results from this study were also compared to those from the other studies using the same dataset.

Several researchers have presented task-based relevant feature selection approaches to improve the performance of fNIRS-based BCIs [36,45,94,95]. Aydin utilized both filter-based and wrapper-based methods to reduce the redundant features and extract useful information [45]. She showed that the wrapper-based approach had a better performance. In the filtering techniques, the features were selected based on the feature relevance to the dependent variable. She did not find any relevance between the feature and the model performance, while the wrapper methods trained a model using a subset of features to verify their usefulness. In some cases, in the filter-based approaches, a threshold value was selected to remove the redundant information. Wrapper-based techniques can take slightly longer to process, but they have shown that they produce an excellent classification accuracy. In this work, a wrapper-based metaheuristic feature selection framework was proposed to enhance the classification performance of fNIRS-based BCIs. The proposed technique and the literature are compared in Table 6. It is evident from the results that the proposed approach had a better classification performance as compared to the others.

In the study, only continuous metaheuristic feature selection approaches were applied. However, different binary metaheuristic feature selection approaches can be explored in the future to improve the classification accuracy and the feature vector size [96,97,98]. This study’s practical application is in the BCI training phase. The model can be trained offline using the selected features that were determined using the presented wrapper-based approach. In the case of online testing, only the selected features were fed to the trained model for a better classification performance.

## 7. Conclusions

In this study, the wrapper-based metaheuristic optimization algorithms were utilized for activity-related feature selection in the fNIRS-based BCI applications. The performance of seven metaheuristic optimization algorithms (i.e., PSO, CSO, the FA, the BA, FPO, the WOA, and GWO) was analyzed using a k-NN-based cost function. The results demonstrated that all of the metaheuristic optimization algorithms significantly improved the classification accuracy and reduced the feature vector size. After extensive training and testing, GWO obtained the highest classification rates of 94.83 ± 5.5%, 92.57 ± 6.9%, and 85.66 ± 7.3% for the MA, MI (left- and right-hand), and four-class (left- and right-hand MI, MA, and baseline), respectively. Furthermore, the statistical analysis (*p* < 0.01) showed that GWO yielded better and more stable results. Therefore, the wrapper-based metaheuristic optimization algorithms were considered helpful for selecting the appropriate activity-related features for robust fNIRS-based BCI applications. In the future, other latest optimization algorithms and binary versions can be applied to measure their performance on fNIRS-based BCI applications.

## Figures and Tables

**Table 1:** Parameters for each algorithm.

PSO	CSO	FA	BA	FPO	WOA	GWO
$\begin{array}{l} T=100\\ N=10\\ c_{1}=1\\ c_{2}=2\\ \alpha =2 \end{array}$	$\begin{array}{l} T=100\\ N=10\\ \alpha =1\\ \lambda =1.5 \end{array}$	$\begin{array}{l} T=100\\ N=10\\ \alpha =1\\ \beta_{0}=1\\ \gamma =1 \end{array}$	$\begin{array}{l} T=100\\ N=10\\ f_{\max}=2\\ f_{\min}=0\\ \alpha =\beta =0.9\\ A=2 \end{array}$	$\begin{array}{l} T=100\\ N=10\\ \lambda =1.5 \end{array}$	$\begin{array}{l} T=100\\ N=10\\ b=1\\ l=1 \end{array}$	$\begin{array}{l} T=100\\ N=10 \end{array}$

**Table 2:** Classification performance of each subject for the mental arithmetic (MA) and baseline tasks in terms of the accuracy (Acc) and the feature vector size (F.V.S.) (data represented as the mean ± the standard deviation).

Subject	PSO		CSO		FA		BA		FPO		WOA		GWO		Full Features	
	F.V.S.	Acc (%)	F.V.S.	Acc (%)	F.V.S	Acc (%)	F.V.S.	Acc (%)	F.V.S.	Acc (%)	F.V.S.	Acc (%)	F.V.S.	Acc (%)	F.V.S.	Acc (%)
1	57.9 ± 8.4	96.6 ± 4.3	64.6 ± 6.1	96.6 ± 5.8	77.6 ± 8.4	97.5 ± 4	72.5 ± 5.9	98.3 ± 3.5	78 ± 3.9	97.5 ± 4	36.1 ± 35.5	94.1 ± 6.8	24.1 ± 5.1	99.1 ± 2.6	180	91.67
2	58.8 ± 12.8	94.1 ± 5.6	59 ± 6.4	90.8 ± 8.2	76 ± 6.4	91.6 ± 10.3	70.6 ± 6.4	89.1 ± 7.9	78.3 ± 7.8	91.6 ± 5.5	6.6 ± 3.9	90 ± 8.6	13.6 ± 5.2	94.1 ± 8.8		91.67
3	56.9 ± 5.8	85.8 ± 11.1	67.9 ± 5.7	85.8 ± 10.4	81.2 ± 7.4	83.3 ± 9.6	74.3 ± 5	78.3 ± 8.9	81 ± 5.8	74.1 ± 13.2	12.5 ± 14	81.6 ± 8.6	21.3 ± 4.6	93.3 ± 6.5		83.33
4	57.6 ± 6.2	93.3 ± 6.5	58.7 ± 4.6	88.3 ± 8.9	75.1 ± 5.7	90 ± 6.5	69.4 ± 5.5	89.1 ± 8.8	75.3 ± 5.6	84.1 ± 7.2	4.8 ± 2.8	88.3 ± 8.9	14.4 ± 4.4	93.3 ± 6.5		91.67
5	64.4 ± 8.7	82.5 ± 13.2	70.9 ± 4.5	82.5 ± 14.4	80.6 ± 6.4	83.3 ± 11.7	80.6 ± 7.1	84.1 ± 13.8	79.3 ± 5.5	83.3 ± 10.3	10.1 ± 14.7	85.8 ± 6.8	27.3 ± 5.8	94.1 ± 5.6		58.33
6	58.8 ± 7.5	85.8 ± 7.9	70.4 ± 7.4	89.1 ± 7.9	80.1 ± 6.5	84.1 ± 9.1	77 ± 6.6	84.1 ± 8.2	79.8 ± 6.6	77.5 ± 13	12.6 ± 14.5	88.3 ± 9.7	22.8 ± 7.6	94.1 ± 8.8		91.67
7	59.9 ± 6.9	84.1 ± 9.1	69.7 ± 9	91.6 ± 7.8	86.2 ± 6.4	87.5 ± 7	76.6 ± 4.7	82.5 ± 9.1	79.6 ± 6.5	84.1 ± 9.1	15.3 ± 29.9	90.8 ± 7.2	27.7 ± 8.1	95.8 ± 7		75
8	63.6 ± 5.3	89.1 ± 6.8	68.6 ± 7	89.1 ± 6.8	80.8 ± 7.8	81.6 ± 10.2	76.1 ± 5.8	84.1 ± 11.4	81.9 ± 5.7	83.3 ± 12.4	17.9 ± 25.8	91.6 ± 7.8	21.6 ± 3.6	94.1 ± 6.8		75
9	63.1 ± 8.2	96.6 ± 5.8	66.4 ± 4.6	95 ± 7	81 ± 4.4	92.5 ± 8.2	79.9 ± 6.2	97.5 ± 4	81.3 ± 3.5	90 ± 6.5	18 ± 20.4	96.6 ± 5.8	20.2 ± 6.2	99.1 ± 2.6		91.67
10	54.2 ± 6.9	100 ± 0	54.4 ± 5.1	100 ± 0	72.3 ± 5	100 ± 0	65.5 ± 3.7	100 ± 0	76.4 ± 5.6	100 ± 0	7.2 ± 7	100 ± 0	6.9 ± 2.8	100 ± 0		100
11	67.5 ± 6.7	90.8 ± 7.2	73.7 ± 6.9	81.6 ± 6.5	81.5 ± 5.2	77.5 ± 5.6	82.3 ± 5.5	88.3 ± 8.9	83.4 ± 8	85 ± 6.5	17.9 ± 27.6	81.6 ± 7.6	28 ± 6	91.6 ± 6.8		75
12	60.6 ± 8.5	85 ± 6.5	62.6 ± 6	80 ± 12.5	84.3 ± 8	80 ± 10.5	77.6 ± 6.5	80.8 ± 7.9	80.1 ± 6.4	75 ± 10.3	5.7 ± 4.1	89.1 ± 5.6	18.8 ± 7.8	93.3 ± 5.2		75
13	66.7 ± 7.5	87.5 ± 14.8	74.2 ± 3.9	85 ± 9.4	82 ± 4.5	80.8 ± 7.9	80.5 ± 5.3	81.6 ± 11.6	79.5 ± 5.9	82.5 ± 9.1	21.9 ± 28.5	86.6 ± 5.8	27.1 ± 5.1	93.3 ± 8.6		66.67
14	66.4 ± 8.9	84.1 ± 10.7	67.4 ± 4.5	87.5 ± 7	83.1 ± 6.4	85.8 ± 5.6	77.9 ± 6.8	78.3 ± 9.7	82 ± 6.6	79.1 ± 9	17.3 ± 21.7	84.1 ± 10.7	23.9 ± 5	94.1 ± 4		83.33
15	71.8 ± 7.2	87.5 ± 9.8	71.7 ± 3.5	77.5 ± 11.8	85.6 ± 7.9	81.6 ± 10.2	82.5 ± 8.8	81.6 ± 7.6	81.6 ± 5.5	78.3 ± 7	17.1 ± 37.2	79.1 ± 5.8	28.9 ± 7.4	94.1 ± 9.6		66.67
16	67.8 ± 7.8	80.8 ± 9.6	72.4 ± 3.6	80 ± 11.9	83.8 ± 5	81.6 ± 14	76 ± 5.7	81.6 ± 9.4	78.4 ± 4.7	77.5 ± 15.2	6.5 ± 10.1	79.1 ± 10.5	26.1 ± 4.6	85.8 ± 8.8		75
17	61.6 ± 5.1	94.1 ± 5.6	67.1 ± 4.4	95.8 ± 5.8	78.3 ± 5	91.6 ± 6.8	78.3 ± 4.6	92.5 ± 6.1	80.7 ± 5.1	90.8 ± 8.2	37.2 ± 40.5	93.3 ± 5.2	17.5 ± 4.7	96.6 ± 4.3		83.33
18	56.8 ± 4.1	95.8 ± 4.3	62.6 ± 8.1	96.6 ± 4.3	81.3 ± 4.4	96.6 ± 5.8	76.3 ± 5.3	93.3 ± 7.6	77.3 ± 7.7	97.5 ± 4	8 ± 9.2	95.8 ± 4.3	12.5 ± 5.7	96.6 ± 4.3		83.33
19	57.8 ± 5.8	86.6 ± 8	70.1 ± 10.2	86.6 ± 11.9	80.2 ± 7.3	90 ± 11.6	77.8 ± 6	86.6 ± 8.9	80.4 ± 5	84.1 ± 9.9	19.8 ± 27.3	89.1 ± 9.6	17.8 ± 6.4	94.1 ± 5.6		83.33
20	61.8 ± 6.9	83.3 ± 10.3	68.1 ± 7.8	81.6 ± 11.6	78.6 ± 7.8	85 ± 10.9	79 ± 6.6	83.3 ± 9.6	84.8 ± 7.8	82.5 ± 8.2	11.3 ± 12.3	85 ± 7.6	23.8 ± 4.7	93.3 ± 6.5		83.33
21	60.5 ± 8.6	90.8 ± 8.2	69.7 ± 6.6	91.6 ± 8.7	86.5 ± 6.3	86.6 ± 5.8	79.6 ± 7.6	90 ± 9.4	79.4 ± 3.2	90.8 ± 10.7	14.1 ± 12.5	93.3 ± 8.6	24.6 ± 5.7	99.1 ± 2.6		100
22	61.1 ± 5.7	90 ± 11.6	73.1 ± 7.6	93.3 ± 6.5	81.9 ± 6.2	85 ± 10.9	79.6 ± 6.6	85.8 ± 13	78.6 ± 7.5	84.1 ± 9.9	14.8 ± 15	89.1 ± 5.6	23.9 ± 6.6	95.8 ± 4.3		83.33
23	65.8 ± 7.5	87.5 ± 11.2	65.4 ± 6.9	88.3 ± 8.9	78.9 ± 5.6	76.6 ± 15.6	77.6 ± 5.1	77.5 ± 15.2	76.9 ± 4.9	77.5 ± 13	16.9 ± 16.4	89.1 ± 7.9	22.1 ± 7.7	94.1 ± 6.8		83.33
24	62.3 ± 5.4	83.3 ± 15.7	70.2 ± 3	85 ± 10.2	81.7 ± 4.9	86.6 ± 8	77.7 ± 4.3	76.6 ± 12.9	82.6 ± 9.1	80 ± 8.9	7.6 ± 12.6	83.3 ± 9.6	26.8 ± 7.2	89.1 ± 4		66.67
25	59.5 ± 10.1	92.5 ± 7.2	68.1 ± 9.8	92.5 ± 9.9	77.2 ± 6.7	90 ± 8.6	79 ± 8.8	93.3 ± 6.5	79.4 ± 5.9	90.8 ± 8.2	23.2 ± 31.1	93.3 ± 5.2	19.4 ± 7.6	99.1 ± 2.6		83.33
26	61.6 ± 6.5	92.5 ± 7.2	65.5 ± 4.4	92.5 ± 7.2	83.1 ± 8.6	88.3 ± 8	79.9 ± 6.1	91.6 ± 6.8	81.6 ± 4.4	88.3 ± 8.9	24.1 ± 25.4	91.6 ± 7.8	22 ± 9.7	94.1 ± 6.8		91.67
27	62.9 ± 7.4	90.8 ± 6.1	73 ± 3	96.6 ± 4.3	78.7 ± 7.2	87.5 ± 8	77.8 ± 8.2	92.5 ± 8.2	82.7 ± 3.4	85.8 ± 7.9	22.3 ± 27.2	91.6 ± 5.5	22.7 ± 6.4	95.8 ± 5.8		83.33
28	60 ± 5.8	86.6 ± 9.7	67.6 ± 7.8	90 ± 11.6	77.3 ± 6.7	89.1 ± 5.6	76.7 ± 6	88.3 ± 7	81.5 ± 7.4	86.6 ± 12.5	9.9 ± 9.8	85 ± 10.2	19.1 ± 4	95 ± 5.8		66.67
29	56.7 ± 7	85 ± 8.6	63.1 ± 9.1	93.3 ± 7.6	78.1 ± 5.7	93.3 ± 6.5	77.1 ± 10.1	90 ± 8.6	79.7 ± 6.9	85.8 ± 13	14.2 ± 14.7	90.8 ± 2.6	22.4 ± 11.4	98.3 ± 3.5		75

**Table 3:** Classification performance of each subject for the left-hand motor imagery (LHMI) and right-hand motor imagery (RHMI) tasks in terms of the accuracy (Acc) and the feature vector size (F.V.S.) (data represented as the mean ± the standard deviation).

Subject	PSO		CSO		FA		BA		FPO		WOA		GWO		Full Features	
	F.V.S.	Acc (%)	F.V.S.	Acc (%)	F.V.S	Acc (%)	F.V.S.	Acc (%)	F.V.S.	Acc (%)	F.V.S.	Acc (%)	F.V.S.	Acc (%)	F.V.S.	Acc (%)
1	69.4 ± 5.5	82.5 ± 9.1	74.8 ± 6.9	76.6 ± 10.2	85.1 ± 6.9	70 ± 8.9	81.4 ± 5.1	70 ± 7	83.2 ± 6.3	70 ± 8	17.5 ± 32.5	77.5 ± 5.6	35.7 ± 13.7	92.5 ± 8.2	180	41.67
2	63.6 ± 4.7	87.5 ± 9	68.7 ± 6.7	94.1 ± 8.8	79.3 ± 6.7	85 ± 7.6	76 ± 5.3	91.6 ± 6.8	79.2 ± 6.5	87.5 ± 5.8	21.1 ± 22.1	89.1 ± 6.8	23.3 ± 7.8	94.1 ± 6.8		91.67
3	66.9 ± 9.2	83.3 ± 11.7	69.1 ± 5.9	80 ± 8	82.9 ± 7.6	80 ± 12.5	85.4 ± 7.9	77.5 ± 9.6	85.2 ± 7.3	78.3 ± 8.9	23.2 ± 43.3	86.6 ± 5.8	24.7 ± 6.5	94.1 ± 5.6		66.67
4	62.6 ± 6.4	83.3 ± 11.1	66.2 ± 6.7	84.1 ± 11.4	81.8 ± 5.9	82.5 ± 10.7	80.2 ± 4.3	87.5 ± 8	80.7 ± 5.6	79.1 ± 13.7	9.1 ± 12	83.3 ± 13	22.4 ± 6.6	92.5 ± 8.2		75
5	66.2 ± 4.1	88.3 ± 8	71.2 ± 7.1	78.3 ± 9.7	77.5 ± 4	80 ± 13.7	81.2 ± 9.6	74.1 ± 13.2	82 ± 5.6	80 ± 8	17.2 ± 16.7	85 ± 5.2	27.8 ± 6.5	90.8 ± 9.1		66.67
6	56.3 ± 7.8	88.3 ± 11.9	61.7 ± 2.9	85.8 ± 7.9	78 ± 7.4	86.6 ± 10.5	75.2 ± 6.3	84.1 ± 8.2	77.4 ± 6.8	80 ± 8	8.1 ± 10.4	90 ± 7.6	16.8 ± 6.4	86.6 ± 7		75
7	59.2 ± 7	90.8 ± 8.2	67.7 ± 5.3	90 ± 9.4	79.9 ± 4.8	86.6 ± 8	78 ± 6.2	86.6 ± 7	82.2 ± 8.9	85.8 ± 7.9	19.8 ± 27.8	82.5 ± 6.1	23.3 ± 6.1	95 ± 5.8		75
8	63.7 ± 7.8	79.1 ± 11.9	69.4 ± 5.7	78.3 ± 7	82.4 ± 9.6	74.1 ± 15.9	75.9 ± 6.1	77.5 ± 13.6	79.5 ± 7.5	81.6 ± 10.9	8.2 ± 7.8	81.6 ± 9.4	24.1 ± 3.9	87.5 ± 9.8		83.33
9	68.4 ± 5.3	83.3 ± 12.4	70.4 ± 7.5	77.5 ± 13.6	82.6 ± 7.2	79.1 ± 8	81.3 ± 4.9	75 ± 11.1	86.6 ± 9.1	73.3 ± 12.9	15.4 ± 12.4	84.1 ± 4.7	23.3 ± 6.7	96.6 ± 5.8		66.67
10	66.8 ± 5.7	86.6 ± 9.7	74 ± 4.6	89.1 ± 6.8	80.2 ± 6.5	82.5 ± 7.2	83.3 ± 6.2	80.8 ± 7.9	81.8 ± 4.2	83.3 ± 9.6	15.3 ± 25.6	84.1 ± 10.7	31.1 ± 10.3	92.5 ± 7.2		83.33
11	61.3 ± 9.6	79.1 ± 9.8	73.6 ± 6	85 ± 9.4	82.3 ± 6.4	79.1 ± 7	80 ± 7.6	80 ± 5.8	83.5 ± 5.6	81.6 ± 10.2	8 ± 9	83.3 ± 8.7	19.5 ± 5.1	91.6 ± 9.6		75
12	63.4 ± 7.1	83.3 ± 7.8	64.7 ± 6.6	76.6 ± 10.9	82.5 ± 7.1	80.8 ± 10.4	75.2 ± 5.3	75.8 ± 14.9	78.4 ± 6.4	80.8 ± 10.4	16.5 ± 20.5	80.8 ± 8.8	23.5 ± 8.7	90 ± 8.6		58.33
13	62.5 ± 5.1	89.1 ± 6.8	73.4 ± 7.5	88.3 ± 8	80.6 ± 6.8	84.1 ± 9.1	79.1 ± 4.5	80 ± 9.7	81 ± 4	80 ± 7	14.9 ± 19.9	85 ± 10.2	25.9 ± 8.6	96.6 ± 4.3		66.67
14	65.8 ± 6.4	83.3 ± 5.5	67 ± 6.2	87.5 ± 7	81.3 ± 5.1	89.1 ± 9.6	80.3 ± 4.1	83.3 ± 9.6	80.5 ± 6.8	85.8 ± 8.8	12.4 ± 19.9	83.3 ± 7.8	27.4 ± 5.8	97.5 ± 4		66.67
15	60.3 ± 7.6	74.1 ± 10.7	70 ± 6.5	80 ± 11.2	86.2 ± 7.2	77.5 ± 9.6	78.3 ± 9.5	88.3 ± 9.7	81.1 ± 3.4	80 ± 11.2	10.8 ± 7.4	85.8 ± 10.4	22.1 ± 5.4	93.3 ± 6.5		66.67
16	58.7 ± 5.4	81.6 ± 15.1	66.5 ± 6.1	83.3 ± 11.1	80 ± 5.6	69.1 ± 6.8	76.6 ± 6.2	80 ± 15.8	78.7 ± 6.1	76.6 ± 12.2	11.1 ± 23.3	79.1 ± 10.5	29.3 ± 16.6	94.1 ± 4		91.67
17	64.8 ± 7.2	82.5 ± 11.4	74.2 ± 5.9	85 ± 13.4	80.5 ± 6.4	80.8 ± 9.6	80 ± 9	75 ± 12.4	82.5 ± 5.7	73.3 ± 7.6	19 ± 25.4	80 ± 8.9	27.8 ± 9.4	91.6 ± 9.6		83.33
18	62.8 ± 5.9	88.3 ± 8	68.3 ± 4.8	87.5 ± 9.8	84.7 ± 8.3	82.5 ± 6.1	79.9 ± 10.4	83.3 ± 8.7	78.3 ± 4.6	75.8 ± 11.4	8.5 ± 10.1	81.6 ± 7.6	26.7 ± 5.3	87.5 ± 7		75
19	70.7 ± 4.5	79.1 ± 13.7	74.4 ± 6.8	72.5 ± 12.4	85.1 ± 7.1	71.6 ± 13.7	75.2 ± 5.2	58.3 ± 15.2	84.9 ± 6.6	64.1 ± 12.4	4.2 ± 3.8	80 ± 5.8	32.8 ± 8.2	86.6 ± 8		41.67
20	67.2 ± 7	87.5 ± 7	71.6 ± 7.8	85 ± 9.4	83.4 ± 9.5	80 ± 8.9	75.9 ± 5.4	83.3 ± 11.1	79.2 ± 4	78.3 ± 10.5	25.8 ± 27.6	88.3 ± 8	25.8 ± 5.3	97.5 ± 4		83.33
21	62.8 ± 10.1	79.1 ± 12.5	74.2 ± 8.1	85.8 ± 10.4	80.7 ± 8.4	80.8 ± 7.9	78.4 ± 5.1	81.6 ± 16.5	79.9 ± 7.3	80 ± 9.7	17.8 ± 21.1	86.6 ± 9.7	29.5 ± 8.2	94.1 ± 7.9		66.67
22	59.4 ± 7.8	80.8 ± 9.6	67.7 ± 5.5	78.3 ± 7	80.7 ± 4.6	75 ± 12.4	74.9 ± 3.9	83.3 ± 5.5	84 ± 7.4	75 ± 8.7	8.1 ± 13.1	82.5 ± 8.2	23.5 ± 7.8	90 ± 5.2		66.67
23	68.3 ± 8.4	88.3 ± 12.5	68.8 ± 7.9	88.3 ± 8	83.6 ± 7.6	85 ± 6.5	75.9 ± 5.5	86.6 ± 5.8	81.6 ± 6.3	85 ± 10.2	20.5 ± 29.8	87.5 ± 8	26.9 ± 10	94.1 ± 5.6		75
24	63.1 ± 8.4	77.5 ± 17.5	69.8 ± 4.8	74.1 ± 13.8	82.1 ± 5.5	74.1 ± 9.1	82.1 ± 7.8	77.5 ± 11.1	77.4 ± 5.4	72.5 ± 11.1	8.7 ± 12.6	79.1 ± 7	23.3 ± 4.2	90 ± 6.5		75
25	63.6 ± 8.9	78.3 ± 5.8	72.8 ± 5.7	84.1 ± 6.1	82.2 ± 6.3	80 ± 7	79.8 ± 10	81.6 ± 5.2	80.6 ± 5.1	69.1 ± 12.4	17.1 ± 12.8	85 ± 6.5	31.3 ± 7.5	91.6 ± 7.8		66.67
26	62.1 ± 5	84.1 ± 8.2	72.3 ± 5.9	82.5 ± 9.1	78.2 ± 5.2	81.6 ± 14	77.3 ± 3.8	87.5 ± 14.8	81.3 ± 5.2	80 ± 8	28.6 ± 30.5	81.6 ± 5.2	30.2 ± 13.6	91.6 ± 11.1		66.67
27	66.9 ± 5.8	84.1 ± 10.7	69.9 ± 5.3	90 ± 6.5	81.6 ± 8.2	83.3 ± 9.6	78.7 ± 4.8	85 ± 10.9	81.9 ± 7.7	84.1 ± 7.2	18.1 ± 28.2	90 ± 8.6	24.4 ± 7.6	96.6 ± 8		75
28	68.7 ± 5.1	82.5 ± 9.1	73 ± 7.5	86.6 ± 7	84.8 ± 6.1	83.3 ± 7.8	78.1 ± 5.4	85 ± 10.2	85.7 ± 8.4	82.5 ± 9.9	12.6 ± 6.5	88.3 ± 9.7	25.7 ± 7.7	93.3 ± 5.2		75
29	64.4 ± 6.1	85 ± 12.2	72.2 ± 9.1	85 ± 9.4	80.2 ± 6.7	82.5 ± 9.9	79.1 ± 4.1	75 ± 10.3	82.2 ± 7.6	75 ± 12.4	24.6 ± 37.1	84.1 ± 9.1	28.1 ± 6	95 ± 5.8		66.67

**Table 4:** Classification performance of each subject for the LHMI, RHMI, MA, and baseline tasks in terms of the accuracy (Acc) and the feature vector size (F.V.S.) (data represented as the mean ± the standard deviation).

Subject	PSO		CSO		FA		BA		FPO		WOA		GWO		Full Features	
	F.V.S.	Acc (%)	F.V.S.	Acc (%)	F.V.S	Acc (%)	F.V.S.	Acc (%)	F.V.S.	Acc (%)	F.V.S.	Acc (%)	F.V.S.	Acc (%)	F.V.S.	Acc (%)
1	81.1 ± 6.1	77.5 ± 7.6	81.3 ± 5.5	74.1 ± 8.7	86.1 ± 5.7	75 ± 5.5	84 ± 5.2	73.3 ± 6.8	86.9 ± 4.3	68.3 ± 4.8	51.6 ± 39.5	69.5 ± 6.8	44.8 ± 7.7	82.9 ± 8.2	180	66.67
2	76.6 ± 8.1	79.5 ± 4.5	77.8 ± 4.1	79.1 ± 7.3	83.6 ± 5.4	72.5 ± 5.9	85.8 ± 7.2	76.6 ± 4.4	86.7 ± 5.6	75 ± 9.8	53.9 ± 37.4	72.9 ± 11.3	35.3 ± 5.5	87.9 ± 5.3		70.83
3	71.8 ± 7.1	75.8 ± 7.2	75.9 ± 6	72.9 ± 8.1	83 ± 4.4	73.7 ± 5.5	83.8 ± 8.4	70 ± 11.7	83.7 ± 4.8	70.4 ± 5.3	71.3 ± 51.8	72.9 ± 5.2	36.3 ± 7	83.7 ± 8.8		58.33
4	72.6 ± 8.2	78.7 ± 4.1	73.9 ± 3.9	77 ± 6.2	83.9 ± 3	72.9 ± 7.6	83.3 ± 7.4	74.5 ± 5.7	84.9 ± 6.1	73.7 ± 3.4	60.8 ± 31.4	71.2 ± 8.2	35.4 ± 9.8	86.2 ± 5.9		66.67
5	78.6 ± 12.6	73.3 ± 5.6	81.4 ± 6	75 ± 6.8	85.9 ± 5.2	67.9 ± 7.3	82.6 ± 6.7	72 ± 5.5	83.5 ± 7.6	67 ± 6.9	42 ± 36.1	73.3 ± 6.5	39.4 ± 9.5	86.6 ± 2.6		58.33
6	69.5 ± 4.8	69.5 ± 7.3	76.9 ± 7.1	68.7 ± 7.4	81.4 ± 6	60.4 ± 10.2	82.1 ± 5.6	67 ± 4.9	82.4 ± 8.7	65 ± 7.6	34.1 ± 25.8	63.7 ± 6.5	35.5 ± 5.8	83.7 ± 6.3		50
7	76.2 ± 8.2	79.1 ± 6.5	76 ± 7.6	85 ± 7.9	82.2 ± 5.4	77.9 ± 5.5	84.5 ± 4.9	72.5 ± 6.8	84.1 ± 6.1	77.9 ± 6.2	43.6 ± 31.8	74.1 ± 5.1	37.9 ± 4	90 ± 5.9		58.33
8	77.9 ± 8.1	65.8 ± 7.5	75.1 ± 6.8	68.3 ± 10.6	85.3 ± 7.4	67.9 ± 5.5	83.1 ± 9	61.6 ± 7	85.2 ± 6.9	61.2 ± 7	20.3 ± 17.5	62 ± 7.9	35.2 ± 4.5	84.5 ± 5.2		62.50
9	79.1 ± 6.6	80 ± 7.2	76.1 ± 3.5	79.5 ± 7.4	84.5 ± 5.7	77.9 ± 9.6	85.1 ± 6	76.6 ± 5.9	85.9 ± 6.2	76.6 ± 6.5	51.8 ± 37.9	75.8 ± 8.2	38.4 ± 5.5	91.6 ± 5.8		75
10	70.9 ± 8	92.9 ± 3.4	75.2 ± 6.7	90 ± 4	83.9 ± 5	88.7 ± 6.8	84 ± 7.9	89.5 ± 5.2	83.7 ± 5.5	88.3 ± 5.1	53.9 ± 27.1	86.6 ± 5.4	33.3 ± 5.8	97.5 ± 2.1		83.33
11	75.6 ± 7.3	71.2 ± 9.9	74.3 ± 5.9	65.4 ± 6.8	84.8 ± 6.4	64.1 ± 9.2	87.6 ± 5	67 ± 4.9	83.9 ± 4.6	67.9 ± 3.4	58.8 ± 44.9	62 ± 10.6	43.7 ± 13.5	76.6 ± 7.9		58.33
12	67.9 ± 8.4	70.4 ± 8.6	71.9 ± 6.5	72.5 ± 11.9	83 ± 4.3	70 ± 8.9	79.3 ± 7.7	70.4 ± 6.6	81.2 ± 6	62.9 ± 7.4	17 ± 15.5	63.3 ± 6.4	30.4 ± 6.5	77 ± 5.6		58.33
13	76.7 ± 6.9	77 ± 8.6	77.9 ± 6.4	71.6 ± 6.4	87.7 ± 5.3	67.9 ± 6.2	81.7 ± 5.7	69.5 ± 7.3	87.3 ± 6	65 ± 5.2	39.8 ± 26.8	69.1 ± 8.3	41.9 ± 6.2	87 ± 7.7		58.33
14	77.2 ± 6.3	77 ± 6.2	75.4 ± 6.5	77 ± 5.6	85 ± 6.2	73.7 ± 7.3	83.9 ± 4.6	75.4 ± 6	84.5 ± 6.3	67.9 ± 8.5	35.2 ± 30.2	70.4 ± 8.2	41.4 ± 11.5	85 ± 7.4		75
15	75.3 ± 4.3	69.1 ± 7.6	78.5 ± 9	68.3 ± 6.2	85.5 ± 6.3	61.6 ± 6.1	86.1 ± 5.1	64.1 ± 5.9	84.9 ± 5	58.7 ± 5.3	54.3 ± 44.4	62.5 ± 6.8	39.1 ± 5.9	78.7 ± 6.6		41.67
16	77 ± 8.3	74.5 ± 6	80.2 ± 6.2	70 ± 6.4	84.3 ± 6.1	64.5 ± 7.4	80.8 ± 5.3	65.8 ± 9.5	85.1 ± 5.5	62.9 ± 10.2	39.8 ± 39.4	65.8 ± 10.9	41.1 ± 6.9	81.6 ± 5.9		66.67
17	77.5 ± 6.6	82 ± 7.6	76.1 ± 6.3	75.8 ± 9.7	83.9 ± 7.1	70 ± 3.8	86.6 ± 7.3	72.9 ± 5.9	86 ± 7	69.1 ± 6.8	60.6 ± 41.1	70.4 ± 4.9	37.4 ± 7.9	89.1 ± 4		58.33
18	76.9 ± 8.4	81.6 ± 10.4	74.8 ± 6.9	80 ± 7	82.1 ± 5	72.5 ± 7.6	84.6 ± 5.6	77.5 ± 7.1	87.2 ± 5.7	72.9 ± 9.2	39.6 ± 26.8	75.8 ± 4.7	33.8 ± 5.8	89.1 ± 6.5		66.67
19	78 ± 5.9	70.8 ± 8.5	77.6 ± 6.1	59.5 ± 7	83.9 ± 5.2	59.1 ± 5.4	82.2 ± 7.3	55.8 ± 7.9	81.8 ± 4.3	55 ± 6.4	19.5 ± 28.5	60.4 ± 9	36.9 ± 6.7	78.7 ± 7.4		50
20	77.1 ± 8	77.5 ± 9	73.5 ± 4.7	72.5 ± 5.6	89.6 ± 5.5	68.3 ± 6.2	82.8 ± 5.8	67.5 ± 7.8	87 ± 5.2	70 ± 7.5	54.7 ± 33.6	72 ± 7.8	37.2 ± 7	85 ± 6.5		62.50
21	78.2 ± 5	80.4 ± 10.2	80.7 ± 6.6	77.9 ± 6.8	86.7 ± 7.8	79.1 ± 5.5	85.4 ± 3.2	74.1 ± 8.7	85.2 ± 6.5	75 ± 5.8	53.5 ± 42.2	76.2 ± 7	40.6 ± 7.3	88.3 ± 5.8		62.50
22	78.1 ± 7.5	78.3 ± 8	79.8 ± 5.7	73.3 ± 6.5	86.2 ± 6.3	72 ± 3.9	85.4 ± 6.8	70.8 ± 4.8	85 ± 3.3	66.6 ± 7	46 ± 25.2	72 ± 7.6	44.1 ± 9.9	83.3 ± 5.8		70.83
23	70.9 ± 5.6	82 ± 9.4	78.2 ± 7.8	82 ± 6.5	83.8 ± 5.9	75.8 ± 8.2	86.2 ± 8.1	78.3 ± 10.1	84.1 ± 7.4	77.5 ± 6.5	52.5 ± 44.6	74.1 ± 5.4	38 ± 13	85 ± 10		75
24	78.4 ± 7.4	75.8 ± 6.1	74.6 ± 4.8	66.2 ± 6.3	84.6 ± 7	70 ± 6.4	85.5 ± 6.5	65 ± 6.5	79.8 ± 6.5	62.5 ± 7	38 ± 35.6	72 ± 7.6	32.4 ± 4.8	80.8 ± 5.6		54.17
25	76 ± 3.6	80.8 ± 5.6	80.8 ± 6.5	70 ± 8.2	87.4 ± 7.1	69.1 ± 10.6	83.1 ± 7.5	65 ± 8.3	84 ± 6.1	63.3 ± 9.9	65.3 ± 26.5	69.5 ± 9.6	41 ± 7.7	86.2 ± 4.8		58.33
26	74.8 ± 4.2	76.6 ± 6.8	78.4 ± 5.5	72.5 ± 8.3	86.1 ± 5.8	68.7 ± 7.9	83.1 ± 5.5	70.8 ± 8.7	82.7 ± 3.6	65.8 ± 5.8	50.2 ± 33.3	70 ± 7.2	37.6 ± 5.7	83.7 ± 5.7		54.17
27	77 ± 8.7	85 ± 7.6	81.1 ± 4.3	75 ± 6.2	83.6 ± 4.4	73.3 ± 8.1	86.1 ± 7.2	70.8 ± 7	80.7 ± 7.9	73.3 ± 8.3	51.5 ± 30.3	72 ± 7	39.7 ± 8.9	82.9 ± 8.4		70.83
28	75.5 ± 7.9	69.5 ± 9	78.7 ± 8	67.5 ± 5.4	82.1 ± 5.7	65 ± 4	85.1 ± 6.6	59.5 ± 9.2	84.2 ± 9	63.7 ± 9.4	37.8 ± 21.6	64.5 ± 7.6	37.8 ± 6.6	78.3 ± 8.2		62.50
29	68.9 ± 3.7	74.5 ± 6	77.2 ± 7.4	74.5 ± 9.3	81.4 ± 6	73.3 ± 6.8	81.5 ± 5.2	75.8 ± 5.8	87.1 ± 5.8	70 ± 7.5	57.4 ± 46	75.4 ± 5.3	33.9 ± 7.5	84.5 ± 9.6		66.67

**Table 5:** Average feature vector size and processing time of the optimization algorithms.

Metaheuristic Algorithm	Feature Vector Size	Processing Time (s)
PSO	67	2.37
CSO	72	4.48
FA	82	10.08
BA	80	2.32
FPO	82	2.27
WOA	26	2.00
GWO	29	2.17

**Table 6:** Comparison of the proposed framework with the studies using the same dataset.

Ref.	MA (%)	MI (%)	Four-Class (LHMI, RHMI, MA, and Baseline) (%)
[36]	88.1	87.2 (LHMI), 88.4 (RHMI)	-
[45]	86.83	77.41	-
[55]	83.6	63.5	-
[94]	84.94	70.14	-
[95]	82.76	65.86	-
Proposed approach	94.83 ± 5.5%	92.57 ± 6.9%	85.66 ± 7.3%

## Data Availability

The data used to support the findings of this study are included within the article.
